# The N3RO trial: a randomised controlled trial of docosahexaenoic acid to reduce bronchopulmonary dysplasia in preterm infants < 29 weeks’ gestation

**DOI:** 10.1186/s12887-016-0611-0

**Published:** 2016-06-01

**Authors:** Carmel T. Collins, Robert A. Gibson, Maria Makrides, Andrew J. McPhee, Thomas R. Sullivan, Peter G. Davis, Marta Thio, Karen Simmer, Victor S. Rajadurai, the N3RO Investigative Team, Philip Ryan, Scott Morris, Michael Stark, Javeed Travadi, Ian Wright, Kenneth Tan, James Holberton, Gillian Opie, Ian Callander, Jacqueline Stack, Doron Shein, Sarah Bellhouse, Srinivas Bolisetty, Kei Lui, Helen Liley, Mary Berry, Deborah Harris, Mei Chien Chua, Pooja Agarwal

**Affiliations:** Women’s and Children’s Health Research Institute, North Adelaide, South Australia Australia; Healthy Mother’s, Babies and Children, South Australian Health and Medical Research Institute, Adelaide, South Australia Australia; Discipline of Paediatrics, School of Medicine, The University of Adelaide, Adelaide, South Australia Australia; School of Agriculture, Food and Wine, The University of Adelaide, Adelaide, South Australia Australia; Neonatal Services, Women’s and Children’s Health Network, Adelaide, South Australia Australia; School of Public Health, The University of Adelaide, Adelaide, South Australia Australia; Newborn Research Centre, The Royal Women’s Hospital, Melbourne, VIC Australia; Department of Obstetrics and Gynaecology, The University of Melbourne, Melbourne, Australia; Clinical Sciences, The Murdoch Children’s Research Institute, Parkville, VIC Australia; PIPER-Neonatal Retrieval Service, The Royal Children’s Hospital, Melbourne, VIC Australia; Centre of Neonatal Research and Education, The University of Western Australia, Perth, WA Australia; King Edward Memorial Hospital and Princess Margaret Hospital for Children, Subiaco, WA Australia; Department Neonatology, KK Women’s and Children’s Hospital, Singapore, Singapore; Yong Loo Lin School of Medicine, National University of Singapore, Singapore, Singapore

**Keywords:** Infant, Preterm, Bronchopulmonary dysplasia, Docosahexaenoic acid, n-3 long chain polyunsaturated fatty acids

## Abstract

**Background:**

Bronchopulmonary dysplasia (BPD) is a major cause of mortality and long-term respiratory and neurological morbidity in very preterm infants. While survival rates of very preterm infants have increased over the past two decades there has been no decrease in the rate of BPD in surviving infants. Evidence from animal and human studies has suggested potential benefits of docosahexaenoic acid (DHA), an n-3 long chain polyunsaturated fatty acid, in the prevention of chronic lung disease. This randomised controlled trial aims to determine the effectiveness of supplementary DHA in reducing the rate of BPD in infants less than 29 weeks’ gestation.

**Methods/design:**

This is a multicentre, parallel group, randomised, blinded and controlled trial. Infants born less than 29 weeks’ gestation, within 3 days of first enteral feed and with parent informed consent are eligible to participate. Infants will be randomised to receive an enteral emulsion containing DHA or a control emulsion without DHA. The DHA emulsion will provide 60 mg/kg/day of DHA. The study emulsions will continue to 36 weeks’ postmenstrual age (PMA). The primary outcome is BPD as assessed by the requirement for supplemental oxygen and/or assisted ventilation at 36 weeks’ PMA. Secondary outcomes include the composite of death or BPD; duration of respiratory support and hospitalisation, major neonatal morbidities. The target sample size is 1244 infants (622 per group), which will provide 90 % power to detect a clinically meaningful absolute reduction of 10 % in the incidence of BPD between the DHA and control emulsion (two tailed α =0.05).

**Discussion:**

DHA supplementation has the potential to reduce respiratory morbidity in very preterm infants. This multicentre trial will provide evidence on whether an enteral DHA supplement reduces BPD in very preterm infants.

**Trial registration:**

Australia and New Zealand Clinical Trial Registry: ACTRN12612000503820. Registered 09 May 2012.

## Background

### Introduction

Bronchopulmonary dysplasia (BPD) is a serious lung disease occurring in infants born prematurely, with those born at less than 29 weeks’ gestation most at risk. BPD is characterised by the need for supplemental oxygen and/or assisted ventilation at 36 weeks’ postmenstrual age (PMA) [1].

Children with BPD are adversely affected through childhood, imposing an extra burden on families, health and education services. In adjusted comparisons involving preterm peers, children who develop BPD have poorer cardio-respiratory outcomes, including reduced airflow, pulmonary hypertension, limited exercise tolerance in later childhood [2, 3], and a greater rate of re-hospitalisation (40–60 vs. 25 %) with longer hospital stays and multiple re-admissions [4]. Such babies also have poorer neurological outcomes than preterm infants without BPD including double the rate of cognitive and motor delay at pre-school age, up to a 1/3 standard deviation lower intelligence quotient at school age, more school-based problems including difficulty with reading, mathematics and spelling, a 50 % greater likelihood of needing educational assistance at school, a greater need for speech therapy (50 vs. 20 %) and an increased incidence of cerebral palsy (15 vs. 3 %) [4]. Reducing BPD would result in savings not only for the health service, but also for families and education services.

In BPD, the lung develops abnormally, with decreased vascular and alveolar development resulting in larger but fewer alveoli. This abnormal lung development results from a process of chronic inflammation [5]. The risk of BPD is inversely related to gestational age. Risk factors that contribute to a pulmonary inflammatory response include perinatal infection, surfactant deficiency, barotrauma, volutrauma and oxygen toxicity [5].

### Docosahexaenoic acid (DHA) supplementation and respiratory function in infants

In infants born at term, several small, randomised controlled trials involving toddlers and children, have shown improved respiratory outcomes in those who consumed formula supplemented with DHA [6–9]. However, there have been no trials specifically designed to determine the effect of DHA supplementation on BPD in preterm infants.

Preterm infants are well recognised as being at risk of DHA insufficiency and have lower blood DHA levels at birth compared with newborn term infants. Early trials involving DHA supplementation of preterm showed improvement in visual function and on this basis all preterm infant formula is now supplemented with DHA at an amount equivalent to that naturally occurring in the breast milk of women consuming a Western diet (0.2–0.3 % of total fatty acids). However, no clinical benefit on BPD was shown in these early trials [10]. Support for a role of DHA in the development of BPD comes from recent observational data showing that a decreased blood DHA level in infants born less than 30 weeks’ gestation was associated with an increased risk of BPD (OR 2.5 95 % CI 1.3 to 5.0) [11].

The best evidence, however, comes from our DINO trial [12]. In the DINO trial the effect on neurodevelopment of breast milk or preterm formula with a DHA concentration of ≈ 1 % of total fatty acids (the estimated in utero accretion rate) was compared with the current standard breast milk or preterm formula DHA concentration (≈0.30 % of total fatty acids) in 657 infants born less than 33 weeks’ gestation [12]. Although the study was not designed to test BPD, an exploratory analysis in the sub-group of infants born less than 29 weeks’ gestational age (*n* = 225), there was a near significant reduction in infants requiring supplemental oxygen at 36 weeks’ PMA (high-DHA 38.6 % vs. standard DHA 51.4 %; RR 0.75, 95 % CI 0.55 to 1.03, *P* = 0.08, unpublished data).

### How might DHA improve outcomes?

The parenteral lipids administered to preterm infants as well as most breast milks and infant formulas are rich in the n-6 fatty acid linoleic acid (LA). This fatty acid acts as a precursor for arachidonic acid (AA) that gives rise to pro-inflammatory eicosanoids and cytokines and also acts as an inhibitor of DHA accumulation in cells. On the other hand, DHA, an n-3 long-chain polyunsaturated fatty acid (LCPUFA), together with eicosapentaenoic acid (EPA) can be incorporated into membrane phospholipids of immune cells, where they serve as precursors for the synthesis of eicosanoids and docosanoids. These in turn can alter cytokine production and modulate immune cell function and suppress inflammatory responses [13–15]. The presence of n-3 LCPUFA lowers the production of inflammatory eicosanoids and cytokines through competition with AA, both as a substrate for cyclooxygenase/lipoxygenase activity, and by inhibiting the conversion of LA to AA [15]. When n-3 LCPUFA are the substrates, less inflammatory mediators are produced. Thus, incorporation of n-3 LCPUFA into lipid membranes and elsewhere may modulate inflammatory activity.

The balance of n-3 LCPUFA and AA is important. For example, an in vitro study using a human lung carcinoma cell line showed that pro-inflammatory cytokine release was reduced with the supply of a high proportion of DHA and was increased with a high proportion of AA [16]. This concept is supported in two preterm infant trials. In our DINO trial [12, 17] the DHA: AA ratio was 2:1, thus favouring the production of anti-inflammatory mediators, and beneficial effects on respiratory function were found in very preterm infants. In the only other trial which used a DHA dose reflective of the estimated in utero accretion rate, DHA was supplemented together with AA in a 1:1 ratio, and a trend towards increased duration of supplemental oxygen and a need for assisted ventilation in the supplemented group were reported [18].

The beneficial effect of DHA on lung function is supported by animal studies. For example, in murine models of hyperoxia-induced lung injury, DHA supplementation reduced inflammatory responses and improved lung growth [19–21]. Separate studies have also demonstrated that exposure to high DHA increased the production of dipalmatoylphosphtidylcholine, the major surfactant lipid in the fetal and neonatal lung [22, 23].

Overall, there are clearly biologically plausible mechanisms by which n-3 LCPUFA, predominantly as DHA, can contribute to reducing the incidence of BPD.

### Method of enteral supplementation

While supplementing lactating women with DHA increases the DHA content of their breast milk, maternal compliance and biological variation, along with the necessary delays in establishing full enteral feeds in this population, means that there is an inconsistent delivery of DHA to the infant. These limitations can be overcome by direct DHA supplementation to the infant, which eliminates the need for maternal compliance or for the infant to be on full enteral feeds in order to receive the target dose.

We have determined that direct enteral DHA supplementation is safe and effective in preterm infants [24]. The oral supplement can be commenced within the first days of enteral feeding and is well tolerated, with no effect on the number of days feeds were interrupted or the time taken to reach full enteral feeds. The dietary DHA level was directly related to blood DHA levels.

### Need for a trial

BPD is a leading cause of mortality and long-term morbidity in infants born less than 29 weeks’ gestational age. As the survival rate of infants less than 29 weeks’ gestational age continues to increase, with no reduction in the incidence of BPD, the clinical and health care burden grows. A randomised controlled trial of the use of DHA as a simple, safe and cost-effective strategy for the reduction of BPD in preterm infants is needed.

### Objectives

The primary objective of this study is to determine the effect of DHA supplementation on the incidence of BPD in infants born less than 29 weeks’ gestational age, assessed by the requirement for supplemental oxygen and/or assisted ventilation at 36 weeks’ PMA, compared with control.

Key secondary objectives of this study include:

- The composite endpoint of death before 36 weeks’ PMA or BPD
- Respiratory support requirements, including the duration of respiratory support, the use of caffeine and the use of postnatal steroids and diuretics for lung disease.
- Severity of BPD
- Feeding tolerance
- Growth
- Length of hospital stay

Other secondary objectives include retinopathy of prematurity, intra-ventricular haemorrhage, cystic periventricular leukomalacia or porencephaly, sepsis, necrotising enterocolitis and death during first hospitalisation.

## Methods

### Trial design

The N3RO trial is designed as a randomised, controlled, clinician, researcher and participant/family blinded, multicentre trial with two parallel groups and a primary outcome of requirement for supplemental oxygen and/or assisted ventilation at 36 weeks’ PMA.

### Participating centres

The sponsoring institution and Trial Coordinating Centre is the Women’s and Children’s Health Research Institute, Adelaide, South Australia. The trial is being conducted in the following 13 perinatal centres:

- **Australia:** Flinders Medical Centre, Adelaide; Women’s and Children’s Hospital, Adelaide; Royal Women’s Hospital, Melbourne; John Hunter Hospital, Newcastle; Monash Medical Centre, Melbourne; King Edward Memorial Hospital, Perth; Mercy Hospital for Women, Melbourne; Liverpool Hospital, Sydney; Royal Hospital for Women, Sydney; Mater Mothers’ Hospital, Brisbane.
- **New Zealand:** Wellington Hospital, Wellington; Waikato Hospital, Hamilton.
- **Singapore:** KK Women’s and Children’s Hospital.

### Study population

Participants are preterm infants born at less than 29 weeks’ gestation.

#### Inclusion criteria

Each infant must meet all of the following criteria to be enrolled in this study:

1. Born at less than 29 weeks’ gestational age.
2. Within 3 days of commencing enteral feeds.
3. Has a legally acceptable representative (parent(s)/guardian) capable of understanding the informed consent document and providing consent on their (the infant’s) behalf.

#### Exclusion criteria

1. Infants who have a major congenital or chromosomal abnormality.
2. Women providing breast milk who are taking supplements providing > 250 mg DHA per day and do not wish to stop taking supplements.
3. Infants participating in another fatty acid study.
4. Infants receiving intravenous lipid emulsions containing fish oil when used as early lipid parenteral nutrition support (e.g. Omegaven, SMOFlipid, Lipoplus).

### Study treatments

Participating infants will be randomised to the DHA emulsion or control emulsion receiving an oral supplement providing either 60 mg/kg/day of DHA or no DHA respectively. The study emulsions are identical in viscosity, colour, packaging and labelling and are iso-caloric.

#### DHA emulsion

The DHA emulsion will be administered to provide 60 mg/kg/day of DHA. The microencapsulated aqueous emulsion of a DHA-rich triglyceride delivers an enteral DHA dose of 60 mg in 0.5mL. The DHA emulsion will be administered in three divided doses of 0.17 mL of emulsion/kg/dose for a total daily volume of 0.5 mL/kg/day.

#### Control emulsion

The control emulsion contains soy oil. The same volume of control emulsion will be given as for the intervention emulsion; i.e. a total daily volume of 0.5 mL/kg/day given in three divided doses of 0.17 mL/kg/dose.

#### Administration

Both the DHA and control emulsions will be administered enterally through the naso/oro-gastric tube immediately preceding a scheduled feed. If the infant’s feeds are stopped the emulsion is also withheld, with administration recommenced as soon as feeds have been restarted.

The study emulsions are ready to administer, requiring no preparation. Storage and dispensing of the study emulsion will be done by the site pharmacists or nominee. The emulsion is to be administered by registered or enrolled nurses according to local practices. The study emulsion will be kept refrigerated and has a shelf life of 9 months. Once opened, the study emulsion will be discarded after 28 days.

The study emulsion will begin as soon as possible after consent and randomisation and will continue until 36 weeks’ PMA or discharge home, whichever occurs first. The local investigator at each centre (or nominee) will prescribe the study emulsion on the infant’s medication chart. The dose calculation will be checked weekly against the current weight of the infant; birthweight is used until birthweight is regained, then current weight used. The investigator (or nominee) will be notified if re-adjustment of the order based on weight is needed.

#### Manufacture of study emulsion

The study emulsions are manufactured in a licensed facility (licensed according to the Code of Good Manufacturing Practice (GMP) and have been donated to the trial by Clover Corporation Limited (Clover Corp), Australia. The emulsions are packaged and labelled in accordance with GMP including individual product ID, batch number, retest date and include the statement “for clinical trial use only”. The study emulsion is supplied as a box of four, 30 mL plastic screw top bottles containing 20 mL of emulsion in each bottle. An inventory is kept of the emulsion supplies at all sites. The emulsion will be labelled N3RO study emulsion and stored refrigerated.

#### Monitoring adherence

The research nurse in each centre will monitor medication charts and record number of doses of emulsion given per day, to ensure compliance.

#### Concomitant care

Apart from the administration of the study emulsion all clinical management of infants enrolled in the N3RO trial will be undertaken under the direction of neonatologists according to local protocols. The use of intravenous lipid emulsions containing fish oil when used as a rescue therapy for Parenteral Nutrition Associated Liver Disease will be at the discretion of the attending neonatologist and local protocols.

### Outcome measures

#### Primary outcome measure

The primary outcome, the incidence of BPD, will be defined on a physiologic basis that combines oxygen and ventilation support with an assessment of oxygen saturation [25]. The diagnosis of BPD will be determined at 36 completed weeks’ PMA (36 weeks and 0 days to 36 weeks and 6 days inclusive) or day of discharge home, whichever occurs first.

Infants receiving endotracheal mechanical ventilation, continuous positive airway pressure [25] or air or supplemental oxygen delivered by a high flow device at ≥ 2 litres per minute [26] will be classified as having BPD without additional testing. For infants on nasal cannula with a flow < 2 litres per minute the “effective” oxygen concentration will be determined using the Benaron-Benitz formula [27]. Infants receiving supplemental oxygen concentration ≥ 30 % with oxygen saturations between 90 and 96 % (inclusive) will be classified as having BPD without additional testing [25]. Infants in supplemental oxygen < 30 % at rest with oxygen saturations ≥ 90 % or oxygen ≥ 30 % with saturations > 96 % [25] or sub-nasal air at < 2 litres per minute will undergo a timed (20 min) stepwise (every 5 min) reduction to room air. Infants failing the reduction phase (oxygen saturation 80% to 89% for 5 consecutive minutes, or < 80% for 15 seconds, or apnoea and/or bradycardia with saturation < 90%) or room air phase  (oxygen saturation < 90% over a 30 minute period) will be classified as having BPD [25]. Infants in room air at the time of the assessment will have a 15 min oximetry assessment. Infants failing this (oxygen saturation  < 90 %) will be classified as having BPD.

#### Secondary outcome measures

A range of standard clinical outcomes for preterm infants < 29 weeks’ gestation are being collected including:

- Death before 36 weeks’ PMA or BPD;
- Respiratory support requirements including the duration of respiratory support from birth to 36 weeks’ PMA and from birth to 40 weeks’ PMA or discharge home whichever occurred first; discontinuation of endotracheal ventilation, non-invasive positive pressure support and low flow or supplemental oxygen to 40 weeks’ PMA or discharge home, whichever occurred first; the use of caffeine and total days caffeine from birth to 40 weeks’ PMA or discharge home, whichever occurred first; the use of postnatal steroids for lung disease ; the use of diuretics for lung disease and total days of diuretic use from birth to 40 weeks’ PMA or discharge home, whichever occurred first; the severity of BPD classified as mild/moderate/severe according to consensus definitions [1]; BPD classified according to supplemental oxygen or any respiratory support at 36 weeks’ PMA or discharge home whichever occurs first, according to clinical management at that time.
- Feeding tolerance assessed by number of days to reach enteral intake ≥ 120 mLs/kg/d and number of days on which one or more feeds were withheld up to the primary outcome.
- Growth – weight, length and head circumference z scores at 36 weeks’ PMA and 40 weeks’ PMA or discharge home, whichever occurred first.
- Deaths before 36 weeks' PMA and during 1^st^ hospitalisation.
- Length of hospital stay to first discharge home.
- Retinopathy of prematurity (stage ≥3), and that requiring treatment.
- Intra-ventricular haemorrhage – any grade and grade III or IV.
- Cerebral cyst formation – including periventricular leukomalacia and porencephalic cysts.
- Sepsis and necrotising enterocolitis.

Clinical data will be collected in accordance with the definitions of the Australia and New Zealand Neonatal Network [28].

### Participant timeline

Infants will receive treatment from enrolment until 36 weeks’ PMA and follow-up will occur until 40 weeks’ PMA or to discharge home, whichever occurs first. At baseline, a 30 μL infant blood sample for fatty acid analysis will be taken, weight, length and head circumference will be measured and baseline clinical and demographic data collected. Weekly compliance data and monthly weight, length and head circumference measurements will be collected. At 36 weeks’ PMA the primary outcome will be assessed, infant weight, length and head circumference will be measured, an infant blood sample (30 μL) will be collected for fatty acid analysis and, if available, a 30 μL breast milk sample (also for fatty acid analysis) will be collected. Secondary outcome data will be collected up to 40 weeks’ PMA or discharge home, whichever occurs first. The date of first discharge home will also be recorded.

### Sample size estimation

The rate of BPD in infants born less than 29 weeks’ gestation in the DINO trial was 51.4 %. To detect a 10 % absolute reduction (19 % relative reduction) in the incidence of BPD at 36 weeks’ PMA from 51.4 to 41.4 % (with 90 % power, two tailed α =0.05) between the DHA emulsion and control emulsion, a sample size of 1244 is required (622 per group). This sample size takes into account the clustering (non-independence) of multiple births (variance inflation factor 11 %) and deaths (4 % as estimated from the DINO trial). The variance inflation factor for multiple births was based on 26 % of infants born less than 29 weeks’ gestational age being from multiple births, an intracluster correlation coefficient for BPD of 0.52 and an average cluster size of 2.07 for these infants (as observed in the DINO trial in which mothers were randomised). While the variance inflation factor may be reduced by randomising infants rather than mothers, the calculated sample size ensures sufficient power even in the unlikely event that all infants from multiple births are randomised to the same treatment arm as their siblings.

### Recruitment

The research nurse will assess infants for eligibility. The parent(s)/guardians of eligible infants will be approached to enter the trial by the local investigator of each centre (or nominee), ideally within 24 to 48 h of birth. If women are in hospital threatening very preterm delivery then, where possible, information about the study will be provided antenatally.

The information sheet will describe the purpose of the study, the procedures to be followed, and the risks and benefits of participation. The investigator, or nominee, will conduct the informed consent discussion and will check that information provided is understood and answer any questions about the study. Consent will be voluntary and free from coercion. Parents willing for their infant to participate will sign a written consent form. A copy of the consent form will be given to the parents and also documented in the infant’s medical record and study Case Report Form. Once consent and the eligibility of the infant is confirmed, the infant will be randomised and the study emulsion assigned.

A record of all infants screened, enrolled and not enrolled will be maintained. Parent(s)/guardians may withdraw their child from the study for any reason at any time. The reason for withdrawal will be recorded. Whenever possible, parent(s)/guardians of infants who are withdrawn from the study will be asked permission to collect follow-up data relevant to the study.

### Randomisation procedures

A computer generated randomisation schedule using balanced variable block design has been generated by an independent statistician who is not involved with trial participants or data analysis. Stratification occurred for sex, study centre and gestational age less than 27 completed weeks’ and 27 to 28 completed weeks’. Infants from multiple births will be randomised individually. The schedule is kept by the independent statistician and the treatment allocation of each randomisation code can be provided to the investigator in case of emergency.

Upon consent, infants are randomised using a customised, purpose built web-based randomisation service. A unique study ID and a study pack with a unique product ID are assigned. The study ID identifies the randomised infant. The product ID identifies a study box containing four bottles of study emulsion, all labelled with the same product ID. Each box of study emulsion will contain either intervention or control emulsion, pre-packed according to the randomisation schedule.

### Blinding

Participants and their family, care providers, data collectors, outcome assessors, research personnel and data analysts will all be blinded to randomisation group. The intervention and control emulsions are identical in viscosity, colour, packaging and labelling and uniquely identified only by a product ID.

#### Emergency unblinding

The randomisation code for an individual participant may only be unblinded in an emergency situation, where the investigator decides a participant cannot be adequately treated without knowing their treatment allocation. The Principal Investigator must be contacted and all attempts to avoid breaking the code (i.e. withdrawal of treatment) should be made. To break the randomisation code the Investigator must contact the randomisation facility/personnel. The time, date, participant number and reason for unblinding must be documented and events leading to the emergency breaking will be recorded.

### Data collection and management

Data are collected by trained research nurses/coordinators at each participating centre onto carbon copy paper Case Report Forms. Data entry and management are coordinated from the Data Management and Analysis Centre (DMAC) at The University of Adelaide under the direction of the study statistician (TRS). There are regular blinded reviews of data quality and data checks using established protocols. Data discrepancy inquiries and responses are managed through a secure web-based management information system.

The Case Report Forms are stored in a locked office at each study site. Only research staff directly involved in the study will have access to the information. Electronic forms of data are stored on secure servers at DMAC. Data are released only to persons authorised to receive those data.

Original/copies of study documents will be retained at each study site or in archives. No paper records are retained by DMAC at study closeout, all are returned to the coordinating centre, the Women’s and Children’s Health Research Institute. Documents will be retained for at least 30 years after study completion in line with the data retention schedules for research involving minors. At the completion of this time documentation will be destroyed using confidential document disposal. The study electronic data will be stored indefinitely on DMACs secure servers with access only granted to authorised study personnel.

### Statistical methods

All participants will be analysed according to the group to which they were randomised (intention-to-treat principle). The incidence of BPD will be compared between groups using a log binomial regression model with a generalised estimating equation used to account for dependence due to multiple births. The difference between the groups will be expressed as a relative risk with a 95 % confidence interval. Adjustment will be made for randomisation strata (study centre, sex and gestational age less than 27 completed weeks and 27 to less than 29 completed weeks). Secondary analyses will use log-binomial regression models for binary outcomes and linear regression models for continuous outcomes, with generalised estimating equations used to account for dependence due to multiple births. Missing data will be multiply imputed under a missing at random assumption. Sensitivity analyses will also be performed using the original unimputed data.

In planned sub-group analyses of the primary and secondary outcomes we will examine the effect of DHA supplementation on boys and girls separately, and on infants with gestational age less than 27 completed weeks or 27 to less than 29 completed weeks separately. Effect modification by these two factors (sex and gestational age) will be assessed separately by including interaction effects in the statistical models. Separate estimates of treatment effect will be obtained for males and females and for infants born less than 27 completed weeks’ gestation and 27 to less than 29 weeks’ gestation, independent of whether the interaction effect is statistically significant, since this is a priori of interest.

### Data monitoring and harms

An independent Trial Monitoring Committee has been set up to review the progress of the trial and provide feedback to the Trial Management Committee. The Trial Monitoring Committee reviews general study progress (recruitment, compliance, loss to follow-up), the BPD event rate and key secondary/safety outcomes. The Trial Monitoring Committee provides advice regarding external issues that may impact on the N3RO trial (for example changes in clinical practice). The committee meets annually or as required and consists of two neonatologists with clinical trials experience, and a statistician with clinical trials expertise.

An independent (blinded) Serious Adverse Event Committee has been established to review serious adverse events. There are no serious adverse events which would be anticipated as a unique consequence of participation in the trial, however all deaths are to be reported. The Serious Adverse Event Committee includes experts in neonatology and pathology who are not involved in the trial. The primary role of the Serious Adverse Event Committee is to review all deaths to determine whether there is any likelihood that involvement in the trial could have contributed to the death. The cause of the death is determined from the autopsy results or other hospital summary of the death by the relevant medical personnel. This committee meets six monthly, or as required.

Any event that in the opinion of an Investigator may be of immediate or potential concern for an infant’s health or well-being is to be reported immediately to the Chair of the Steering Committee. Expected events such as necrotising enterocolitis, BPD, intracranial abnormality, pulmonary haemorrhage and patent ductus arteriosus are captured by data collection in the Case Report Form. These data are available for review by the Trial Monitoring Committee.

### Research ethics approval

This protocol and the informed consent document have been approved by the Human Research Ethics Committee (HREC) of each study site. Australia: Southern Adelaide Clinical HREC (Coordinating HREC and Flinders Medical Centre, Adelaide); Women’s and Children’s Health Network HREC (Women’s and Children’s Hospital, Adelaide); The Royal Women’s Hospital HREC (Royal Women’s Hospital, Melbourne); The Southern Health HREC (Monash Medical Centre); Mercy Health HREC (Mercy Hospital for Women, Melbourne); Women and Newborn Health HREC (King Edward Memorial Hospital, Perth); Hunter New England HREC (John Hunter Hospital, Newcastle; Liverpool Hospital, Sydney; Royal Hospital for Women, Sydney; Mater Mothers’ Hospital, Brisbane). New Zealand: Northern B Health and Disability HREC (Wellington Hospital, Wellington; Waikato Hospital, Hamilton). Singapore: SingHealth Centralised Institutional Review Board E (KK Women’s and Children’s Hospital).

Any subsequent modifications will be reviewed and approved by the HREC of each study site. The study will be conducted in compliance with the current version of the protocol. Any change to the protocol document or informed consent form that affects the scientific intent, study design, patient safety, or may affect a participant’s willingness to continue participation in the study will be considered a major amendment. All such amendments will be submitted to the HREC for approval. Any other changes to the protocol (such as administrative changes to dates and study personnel) will be considered minor amendments and will be notified to the HREC as appropriate.

### Confidentiality

Participant confidentiality is strictly held in trust by the participating investigators and research staff and their agents. This confidentiality is extended to cover testing of biological samples in addition to the clinical information relating to participants. The study protocol, documentation, data and all other information generated will be held in strict confidence. No information concerning the study or the data will be released to any unauthorised third party, without prior written approval of the Coordinating Centre. The Coordinating Centre and regulatory authorities may inspect all documents and records required to be maintained by the Investigator, including but not limited to, medical records and pharmacy records for the infants in this study subject to individuals having obtained approval/clearance through State/National Governments and HREC as required by local laws. The study site will permit access to such records. Clinical information will not be released without written permission of the parent/guardian, except as necessary for monitoring by HREC or regulatory agencies.

## Discussion

This protocol describes a randomised controlled trial comparing the effect of an enteral emulsion providing supplementary DHA (60 mg/kg/day) with a control enteral emulsion containing no DHA on the incidence of BPD in preterm infants born < 29 weeks’ gestation. BPD is a serious complication of preterm birth, associated with long-term morbidity. Both animal and human studies point to the possibility that DHA may have a primary role in the prevention of BPD however there have been no randomised controlled trials designed specifically to determine the effect of DHA on BPD. To the best of our knowledge this is the first such randomised controlled trial. Results from this study will provide the first high quality evidence on whether an enteral DHA supplement reduces the incidence of BPD in very preterm infants.

## Abbreviations

AA, arachidonic acid; BPD, bronchopulmonary dysplasia; DHA, docosahexaenoic acid; DINO, the acronym is derived from ‘**D**ocosahexaenoic Acid (DHA) for the **i**mprovement of **n**eurodevelopmental **O**utcomes’ in Preterm Infants; DMAC, data management and analysis centre; EPA, eicosapentaenoic acid; GMP, code of good manufacturing practice; HREC, Human Research Ethics Committee; LA, linoleic acid; LCPUFA, long-chain polyunsaturated fatty acid; N3RO, the acronym is derived from ‘**N-3** fatty acids for improvement in **R**espiratory **O**utcomes’; NHMRC, National Health and Medical Research Council of Australia; PMA, postmenstrual age
